# Distribution of *Alternaria* toxins in tomato pulp and peel and their stability to heat treatments

**DOI:** 10.3389/ffunb.2025.1516557

**Published:** 2025-06-06

**Authors:** Paola Giorni, Erica Barato, Terenzio Bertuzzi

**Affiliations:** ^1^ Department of Sustainable Crop Production (DIPROVES), Faculty of Agricultural, Food and Environmental Science, Università Cattolica del Sacro Cuore, Piacenza, Italy; ^2^ Department of Animal Science, Food and Nutrition (DIANA), Faculty of Agricultural, Food and Environmental Sciences, Università Cattolica del Sacro Cuore, Piacenza, Italy

**Keywords:** tomato, *Alternaria* spp., *Alternaria* toxins, tenuazonic acid, alternariol, tentoxin, heat treatments, early blight

## Abstract

**Introduction and methods:**

The distribution of tenuazonic acid (TeA), alternariol (AOH), alternariol monomethyl ether (AME), and tentoxin (TEN) between the pulp and peel was determined in different tomato varieties after artificial inoculation with three *Alternaria* species (*Alternaria alternata*, *Alternaria tenuissima*, and *Alternaria solani*) and incubation for 3 weeks. The role of heat treatments, similar to pasteurization, in their stability was also investigated.

**Results and discussion:**

Unlike AME that was never detected, TeA, AOH, and TEN were determined at different levels in the pulp and peel. Specifically, AOH remained mainly in the peel, where the inoculation was carried out, while TeA and TEN migrated into the pulp and were also found in the discarded liquid accumulated during the incubation period. Heat treatments reduced TeA, AOH, and TEN to varying degrees. In particular, the TeA level was slightly reduced after treatment both at 100°C (approximately 10%) and 121°C (approximately 20%), while a reduction of approximately 30% was achieved after the double heat treatment (treatment at 100°C followed by treatment at 121°C). AOH was found to be less stable to heat treatments, showing a reduction of around 50% after treatment at 100°C and up to 80% after double heating treatments. TEN was reduced by approximately 50% only after the combined treatment of 100°C + 121°C.

## 1 Introduction

*Alternaria* toxins are secondary metabolites produced by fungi that can contaminate cereals, oilseeds, fruits, and vegetables. Their occurrence in tomatoes and derived products has been reported by several authors (da Motta and Soares, 2001; Noser et al., 2011; Bertuzzi et al., 2021; Sanzani et al., 2019; Qin et al., 2022; Ji et al., 2023; Maldonado Haro et al., 2023). In 2016, the European Food Safety Authority (EFSA) published a scientific report evaluating dietary exposure to the main *Alternaria* toxins—alternariol (AOH), alternariol monomethyl ether (AME), tenuazonic acid (TeA), and tentoxin (TEN)—in the European population (EFSA et al., 2016). In this report, “Tomatoes and tomato-based products” resulted as the main contributors to the dietary exposure to TeA for all age classes, with the only exception of the “Infants” and “Toddlers” classes. Successively, the European Commission published the Recommendation 2022/553 reporting on the indicative limits for AOH, AME, and TeA in certain foods including tomato-derived products. AOH and AME have the ability to induce cell cytotoxicity, interfere with the cell cycle, and cause cell apoptosis. Moreover, they can trigger mutagenicity in either bacterial or mammalian cells and are known to induce primary DNA damage *in vitro* and *in vivo*. TeA has been shown to inhibit newly formed proteins from ribosomes, resulting in reduced cell viability across mammalian cell lines. The cytotoxicity effects of TeA involve liver damage, gastrointestinal hemorrhage, cardiovascular collapse, and mortality in various animal species. The toxicological effects of TEN include induction of necrosis, reduction of cell vitality, and interference with plant seedlings (Zhang et al., 2025).

Tomatoes are extremely susceptible to fungal decay due to their thin peel. *Alternaria* spp. are considered among the main causes of black mold disease in raw tomatoes. During industrial tomato processing for the production of several derived products (i.e., ketchups, purées, juices, and sauces), tomatoes are subjected to several immersions in water and to optical and manual selections in order to discard defective tomatoes. Water immersions occur before entering the production plant as a washing step and after the optical and manual selection for easy transport of the tomatoes to the following processing steps. It is unknown whether, during these immersions, *Alternaria* toxins could migrate from contaminated fruits to the water and whether the water, if not frequently replaced, could become contaminated. Successively, different heat treatments are carried out, such as for separation of the skin peel from the pulp, in order to obtain cold or hot break products (for the inhibition or inactivation of pectolytic enzymes, respectively) and to produce concentrated tomato paste. To our knowledge, there are no reports on the distribution of *Alternaria* toxins between the peel and the pulp, while a few studies have indicated their thermal stability during food processing. Estiarte et al. (2018) indicated that AOH and AME are stable during the heat processing of tomatoes, while Siegel et al. (2010) reported stability during bread baking. No results have been reported on the fate of TeA, the main *Alternaria* toxin present in tomatoes, and TEN during tomato processing.

Considering the limited information available on the distribution of *Alternaria* toxins during tomato processing and in the waste and finished products, this study aimed to evaluate the distribution of TeA, AOH, AME, and TEN between the pulp and the peel in artificially contaminated tomatoes and their stability to heat treatments at conditions similar to those used during industrial tomato processing.

## 2 Methods

### 2.1 *Alternaria* spp. inoculum preparation

All of the *Alternaria* strains used for the experiment were purchased from the Westerdijk Fungal Biodiversity Institute (Utrecht, the Netherlands). In particular, one strain of *A. alternata* (CBS 118814), one strain of *A. solani* (CBS 109157), and one strain of *A. tenuissima* (CBS 117.44) were singularly inoculated on Petri dishes (diameter, 9 cm) with potato dextrose agar (PDA; Biolife, Milan, Italy) and incubated at 25°C for 7 days (12-h light/12-h dark photoperiod). After incubation, the developed fungal colonies were washed with sterile water and the spores were collected and mixed together to prepare the inoculum. The concentration of the inoculum was adjusted to ^–^10^4^ CFU/ml.

All of the fungal strains used were tested in previous trials and were shown to be capable of producing the different *Alternaria* toxins: TeA, AOH, AME, and TEN (Bellotti et al., 2023). Different strains of *Alternaria* were considered for the inoculum to better represent the field situation, where more strains are present and able to infect plants. *A. alternata*, *A. tenuissima*, and *A. solani* were chosen as they are more frequently isolated in Italy (Sanzani et al., 2021). Only one strain for each *Alternaria* species was used for the preparation of the artificial inoculum, although the use of more strains would be more representative of species variability. However, this was not crucial to the aim of this study, where only the strains definitely able to produce *Alternaria* toxins to obtain high contamination were used.

### 2.2 Artificial fungal inoculation of tomatoes

A total of 23 samples belonging to seven different tomato varieties were used for the experiment (**Table 1**). These samples are from some of the most cultivated varieties in Northern Italy, i.e., the most significant area in Italy for the production of tomato-based products. The number of samples for each variety was based on the incidence of the presence of each variety in the area.

**Table 1:** Tomato varieties used for artificial inoculation with *Alternaria* species.

Variety	No. of samples
Heinz 1301	10
Heinz 1879	3
Heinz 2123	2
Heinz 3402	2
Heinz 3406	2
NUN 507	2
UG 16112	2

For each sample, approximately 500 g of tomatoes (corresponding to six to eight tomatoes) was placed on a grid in a sterile glass box and artificially inoculated by spraying them with 5 ml of the fungal inoculum, obtained as previously described. To facilitate the fungal inoculation, the peels were wounded at different points with a sterile needle, and 100 ml of sterile water was placed on the bottom of the boxes to maintain high humidity in the chamber. The water on the bottom of the box was kept separate from the tomatoes through a sterile grid and was maintained for the whole incubation period. The boxes were incubated under controlled conditions at 25°C for 21 days (12-h light/12-h dark photoperiod). This incubation time was decided on the basis of a previous *in vitro* experiment conducted with *Alternaria* strains and aimed to define the production of *Alternaria* toxins (Meng et al., 2021; Bagi et al., 2022).

At the same time, 500 g of the same samples was placed in similar boxes, incubated in the same conditions but without artificial fungal inoculation and used as untreated samples for comparison of the results. As all of the tomato samples used were collected from the field at harvest time, possible wounds could have been present on the skin of the untreated samples, as well as possible natural contamination. This could be useful to verify possible differences between natural and artificial contamination in the distribution of *Alternaria* toxins.

### 2.3 Sample preparation and heat treatments

After 3 weeks of incubation, in both inoculated and control samples, the peel was manually separated from the pulp. The peel was approximately 2% of the entire tomato samples. For each sample, the peels obtained from all tomatoes were mixed together to improve homogeneity and used for the analysis. The same procedure was also carried out for the pulp. During incubation, infected tomatoes released liquid due to the decay of the fruits (approximately 10 ml), which mixed with the water present on the bottom of box, which was also collected at the end of incubation.

Subsequently, the pulps and peels were homogenized with UltraTurrax (T25, Janke & Kunkel, Labortechnik, Staufen, Germany) before analysis. The volume of water inside the boxes was measured and then analyzed for the presence of *Alternaria* toxins.

For the control samples, the two parts (peel and pulp) were analyzed for *Alternaria* toxins (TeA, AOH, AME, and TEN). For the artificially inoculated tomatoes, samples of the peel and pulp were divided into four subsamples and were treated following four different treatments: 1) no heating; 2) heating at 100°C for 10 min; 3) heating at 121°C for 20 min; and 4) heating at 100°C for 10 min, followed by 121°C for 20 min. Although this last double treatment is not normally used in the tomato transformation industry, it was included to determine whether longer and stronger heating treatments could have more effect on *Alternaria* toxins.

After cooling, both peel and pulp samples were analyzed for *Alternaria* toxins.

### 2.4 Analysis of *Alternaria* toxin determination

After homogenization using UltraTurrax (16,000 × *g* for 2 min), a volume of 100 ml acetonitrile/water (80 + 20, *v*/*v*) was added to 20 g of the sample to extract mycotoxins using a rotary shaker for 60 min (120 rpm), as reported in our previous work (Bertuzzi et al., 2021). The extract was centrifuged at 4,000 rpm, and then 0.2 ml was diluted with 0.8 ml water/acetonitrile (90:10, *v*/*v*) in a vial for LC-MS/MS (Vanquish pump and autosampler coupled with a Fortis mass spectrometer; Thermo Fisher Scientific, San Jose, CA, USA) analysis in selected reaction monitoring (SRM) mode. *Alternaria* toxins were chromatographed on an HSS-T3 RP-18 column (5-µm particle size, 150 × 2.1 mm; Waters, Milford, MA, USA) using a mobile-phase gradient acetonitrile–water (both acidified with 0.2% formic acid) from 30:70 to 65:35 in 6 min, then isocratic for 3 min; gradient to 30:70 in 1 min and isocratic for 6 min (equilibration step). Ionization was carried out with an electrospray ionization (ESI) interface (Thermo-Fisher, San Jose, CA, USA) in positive mode as follows: spray capillary voltage, 4.5 kV; sheath and auxiliary gas, 35 and 14 psi; and temperature of the heated capillary, 270°C. For fragmentation of the [M]^+^ ions (198 *m*/*z* for TeA, 259 *m*/*z* for AOH, 273 *m*/*z* for AME, and 415 *m*/*z* for TEN), the fragment ions were: 125, 139, and 153 *m*/*z* (16 V) for TeA; 128, 185, and 213 *m*/*z* (35 V) for AOH; 128 and 184 *m*/*z* (38 V) and 258 *m*/*z* (30 V) for AME; and 132 *m*/*z* (37 V) and 135 and 312 *m*/*z* (25 V) for TEN. Quantitative determination was performed using the LC_Quan 2.0 software.

To investigate the matrix effect (ME), the slopes of the matrix-matched calibration curves obtained from five spiked blank sample extracts (four replicates for each sample) were compared with those of the solvent-based calibration curves, calculating ME using the following formula: ME (%) = (slope matrix-matched standard curves/slope solvent standard curves) × 100%. The results showed ME values consistently inferior to 11%. Results inferior to ±15% were generally considered as unaffected by the matrix. The limit of detection (LOD) and the limit of quantification (LOQ) were determined using the signal-to-noise approach, defined as those levels resulting in signal-to-noise ratios of 3 and 10, respectively. The analytic response and the chromatographic noise were both measured from the chromatogram of a blank sample extract to which an appropriate volume of the AOH standard solution had been added. The LOD and the LOQ were 5 and 15 µg/kg for TeA, 0.5 and 1.5 µg/kg for AOH and TEN, and 1 and 2 µg/kg for AME, respectively. The linearity of the calibration curves was established through five calibration standards in solvent, showing *r* values superior to 0.998 (**Supplementary Table S1**). The concentration levels were 25, 50, 100, 250, and 500 µg/L for TeA and 1, 2.5, 5, 10, and 25 µg/L for AOH, AME, and TEN. Recovery values were determined by spiking the aliquot of an uncontaminated (blank) tomato sample (20 g) with an appropriate volume of the AOH standard solution at three different levels before the extraction. Three replicates were analyzed for each level. The recovery values were between 87.3% and 103.1%. The results were corrected for the mean recovery value.

### 2.5 Calculation of TeA and TEN migration in water

The percentage of migration of TeA and TEN in the water added in the inoculation box was calculated as: mycotoxin amount in the measured volume of water solution/(mycotoxin amount in 490 g pulp tomato + amount in 10 g peel (2% of entire tomato)) × 100).

### 2.6 Data analysis

Analysis of variance (ANOVA) on the main factors considered (heat treatments and the distribution of mycotoxins in the fruit) was performed using data from all the samples, despite being from different tomato varieties, and considering them as biological replicates for the statistical analysis. The generalized linear model (GLM) procedure of the IBM SPSS Statistics 27 package (IBM Corp., Armonk, NY, USA) was used. Significant differences were highlighted using Tukey’s test (*p* ≤ 0.05) for mean separation.

Data on *Alternaria* toxin production were logarithmically transformed prior to statistical analysis (values +1). Log transformation is generally required for data that cover a wide range from single-digit numbers to numbers in hundreds or thousands (Clewer and Scarisbrick, 2001), such as those obtained for mycotoxin production.

Moreover, data of the presence of *Alternaria* toxins were analyzed using box plots in order to compare the distribution of data among the different groups (tomato varieties) and to verify consistency of the results obtained.

## 3 Results

In this section, data are presented as value ± standard deviation (SD).

### 3.1 Distribution of *Alternaria* toxins between the pulp and peel

TeA, AOH, and TEN were detected in almost all of the artificially inoculated samples, while AME was never detected in any of them. All of the control samples resulted free from any of the considered *Alternaria* toxins. For this reason, we did not perform thermal treatments on these samples.

Differences in the contamination of tomatoes were determined between the pulp and peel, independently of the variety. The ANOVA results among all the tomato samples used in the experiment showed that the distribution of mycotoxins between the pulp and peel was statistically different for both AOH (*p* ≤ 0.01) and TEN (*p* ≤ 0.05) (**Table 2**).

**Table 2:** Analysis of variance (ANOVA) of the content of *Alternaria* toxins in the peel and pulp of the 23 tomato samples artificially inoculated with *Alternaria* species.

Alternaria toxins	TeA	AOH		TEN	
Fruit section	n.s.	**		*	
Peel	969.7	9.8	A	11.7	A
Pulp	889.6	0.7	B	9.9	B

Different letters indicate significant differences according to Tukey’s test (*p* < 0.01).

*TeA*, tenuazonic acid; *AOH*, alternariol; *TEN*, tentoxin; *n.s.*, not significant.

*p < 0.05, **p < 0.01.

**Figure 1:**
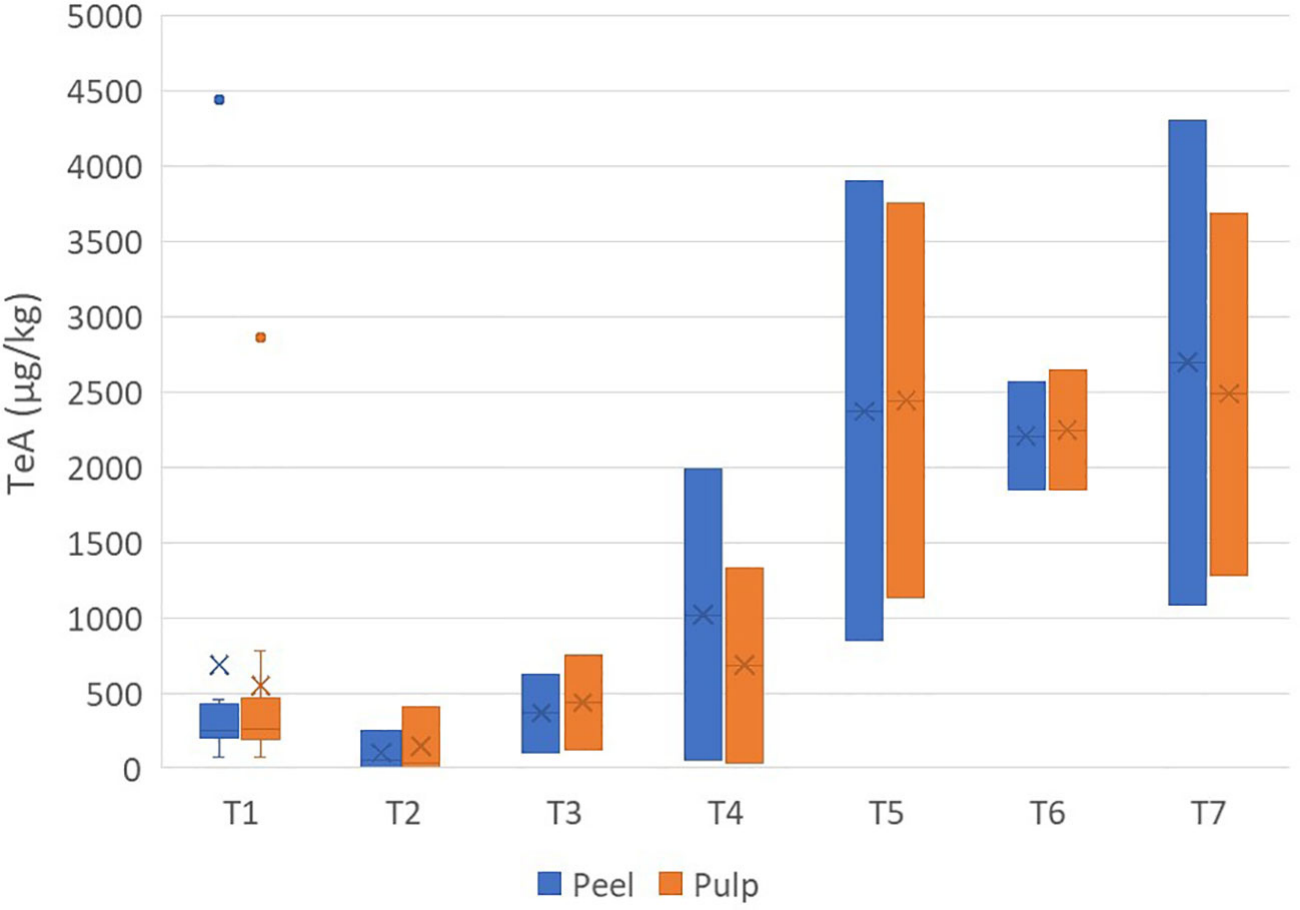
Box plot analysis of the quantity of tenuazonic acid (in micrograms per kilogram) in the peel and pulp of seven different varieties of tomatoes artificially inoculated with *Alternaria* species. Within each box, *horizontal black lines* indicate median values, *crosses* indicate mean values, *boxes* extend from the 25th to the 75th percentile of each group’s distribution of values, the *lower* and *upper lines* denote error lines, and *dots* denote observations outside the 10th and 90th percentiles. The number of samples considered for each tomato variety is reported in **Table 1**.

The concentrations of TeA ranged from 30 µg/kg (pulp sample) to 4,438 µg/kg (peel sample) (**Supplementary Table S2**). The distributions between the peel and pulp were similar, with an average concentration ratio of peel/pulp of 1.09 ± 0.36 (**Figure 1**).

**Figure 2:**
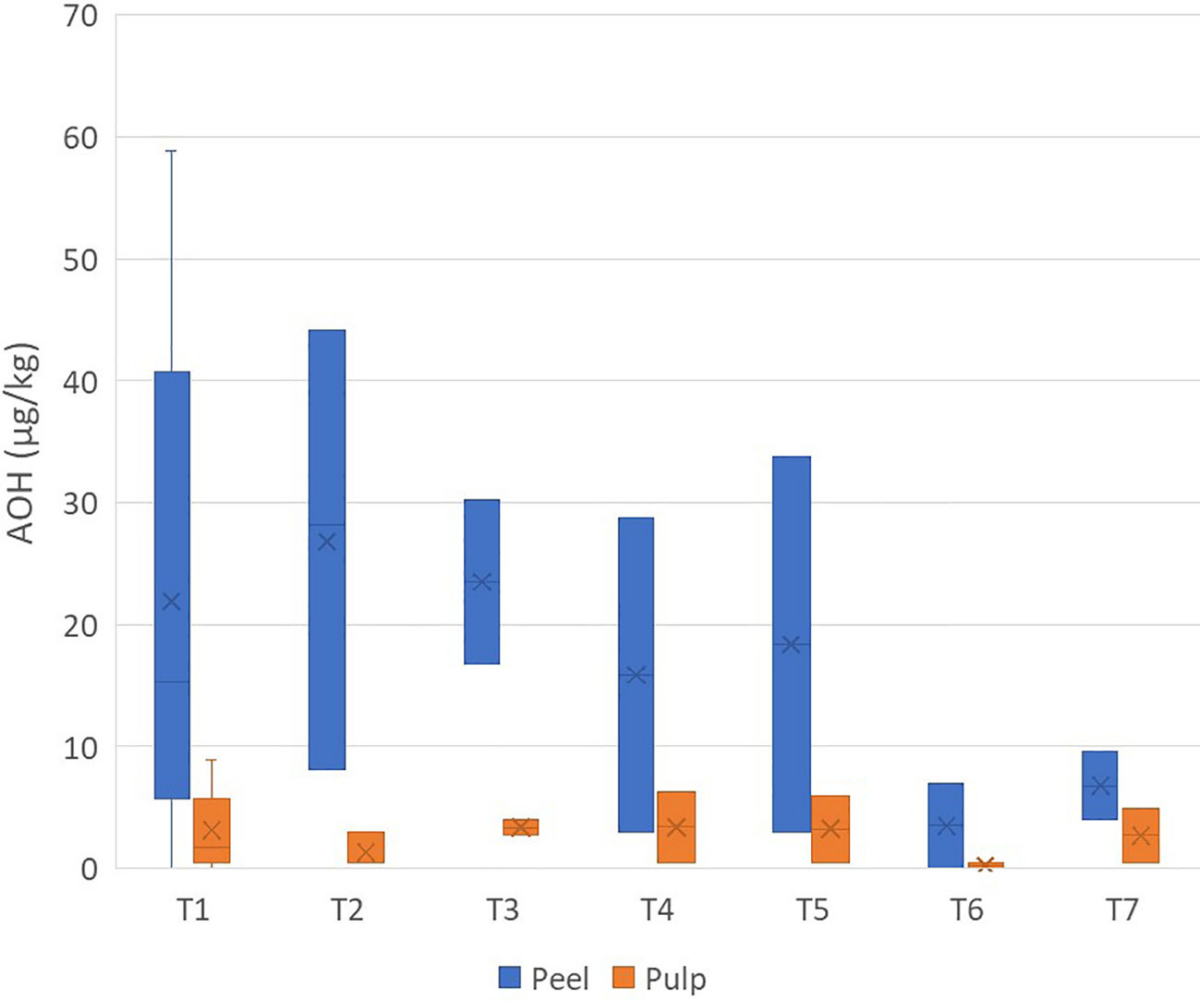
Box plot analysis of the quantity of *Alternariol* (in micrograms per kilogram) in the peel and pulp of seven different varieties of tomatoes artificially inoculated with *Alternaria* species. Within each box, *horizontal black lines* indicate median values, *crosses* indicate mean values, *boxes* extend from the 25th to the 75th percentile of each group’s distribution of values, the *lower* and *upper lines* denote error lines, and *dots* denote observations outside the 10th and 90th percentiles. The number of samples considered for each tomato variety is reported in **Table 1**.

Contamination by AOH was much lower than that by TeA. AOH showed a maximum value of 58.8 µg/kg in a peel sample (in two samples, it was not detected), while only 10 samples of pulp showed contamination (maximum value, 8.0 µg/kg) (**Supplementary Table S3**). Interestingly, AOH occurred mainly in the peel than in the pulp, with the average concentration ratio of peel/pulp being 12.50 ± 13.41 (**Figure 2**).

**Figure 3:**
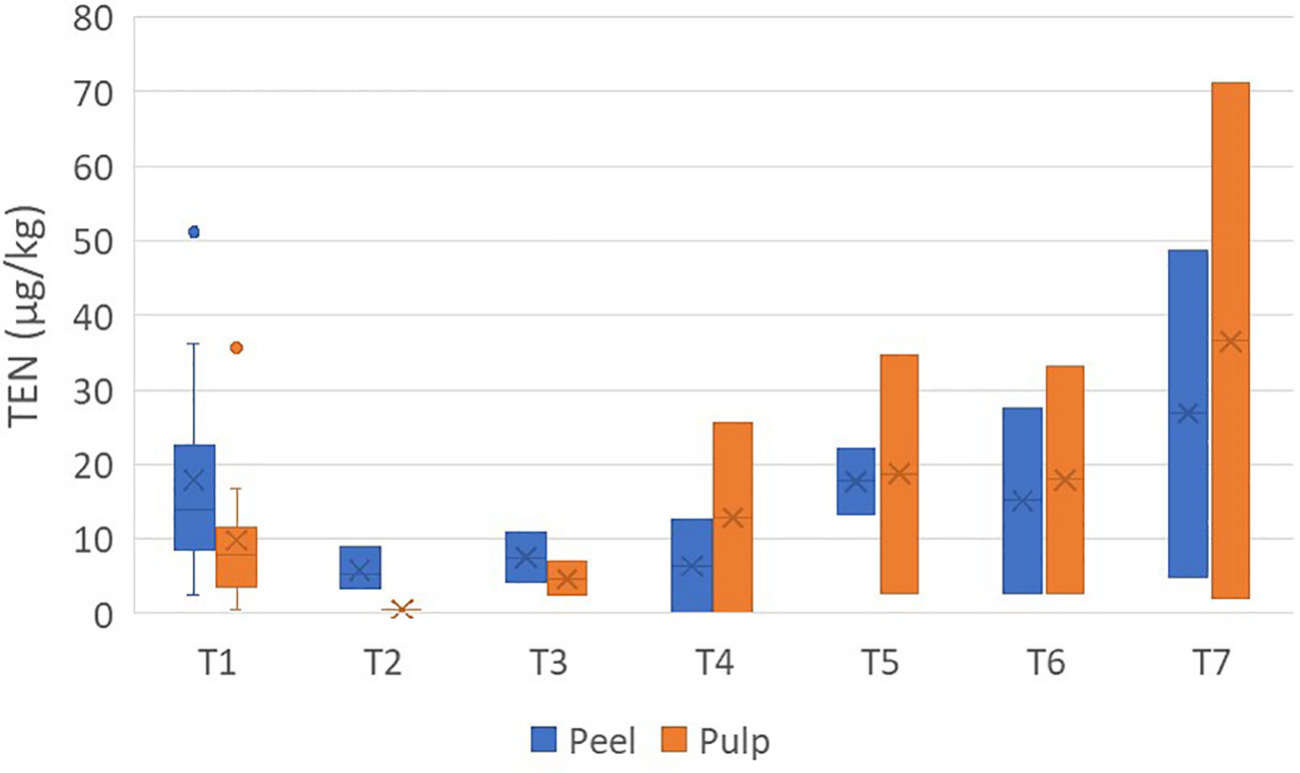
Box plot analysis of the quantity of tentoxin (in micrograms per kilogram) in the peel and pulp of seven different varieties of tomatoes artificially inoculated with *Alternaria* species. Within each box, *horizontal black lines* indicate median values, *crosses* indicate mean values, *boxes* extend from the 25th to the 75th percentile of each group’s distribution of values, the *lower* and *upper lines* denote error lines, and *dots* denote observations outside the 10th and 90th percentiles. The number of samples considered for each tomato variety is reported in **Table 1**.

The concentrations of TEN were found in the range <0.5–71.1 µg/kg. It was not detected in one sample of peel and in five samples of pulp (**Supplementary Table S4**). The average concentration ratio of peel/pulp was 3.83 ± 5.17 and showed a non-constant migration in the pulp (**Figure 3**).

As reported in several studies, TeA is the main toxin detected in tomato products. Our results confirmed this trend. To evaluate whether the level of artificial contamination carried out in the laboratory could be similar to those occurring in the field, a small survey on *Alternaria* toxins was carried out, which sampled naturally contaminated tomatoes from different truck trailers during the unloading stage at an industrial tomato plant. Moldy tomatoes selected by the operators of the plant were collected from nine different lots on different days. Contamination of *Alternaria* toxins was similar to those obtained from the artificial infection carried out in this study. TeA showed a contamination level from <5 to 16,414 µg/kg, AOH from <0.5 to 12 µg/kg, AME from 1 to 4 µg/kg, and TEN from <0.5 to 5.5 µg/kg. In all finished tomato products produced by the industrial plant on the same day of the sampling, TeA was consistently below 160 µg/kg, while AOH, AME, and TEN were never detected.

### 3.2 *Alternaria* toxins in water

A non-negligible migration of TeA and TEN was found in the water added in the incubation boxes. After 3 weeks of incubation, the water also contained liquid released by the moldy tomatoes due to the natural decay of fruits. **Table 3** shows the percentage migration for each variety. The values varied between 9.3% and 26.8% for TeA and between 2.7% and 10.0% for TEN, showing global average percentage migration of 17.7% ± 10.9% and 7.62% ± 6.64% for TeA and TEN, respectively.

**Table 3:** Percentage migration of tenuazonic acid (TeA) and tentoxin (TEN) in water added in the incubation boxes to maintain high levels of humidity.

Tomato variety	Tenuazonic acid (%)	Tentoxin (%)
T1	14.1	9.1
T2	26.8	5.0
T3	21.5	9.7
T4	26.5	9.8
T5	9.3	5.4
T6	11.9	10
T7	20.3	2.7
Mean ± SD	17.7 ± 10.9	7.6 ± 6.6

On the other hand, AOH was never detected in these solutions. Nevertheless, a direct contact does not occur between the water and the tomatoes during the incubation period. These data showed that, differently from AOH, TeA and TEN could easily migrate from the contaminated tomatoes during the washing and movement steps in industrial tomato processing.

### 3.3 Heat stability of TeA, AOH, and TEN

**Figure 4:**
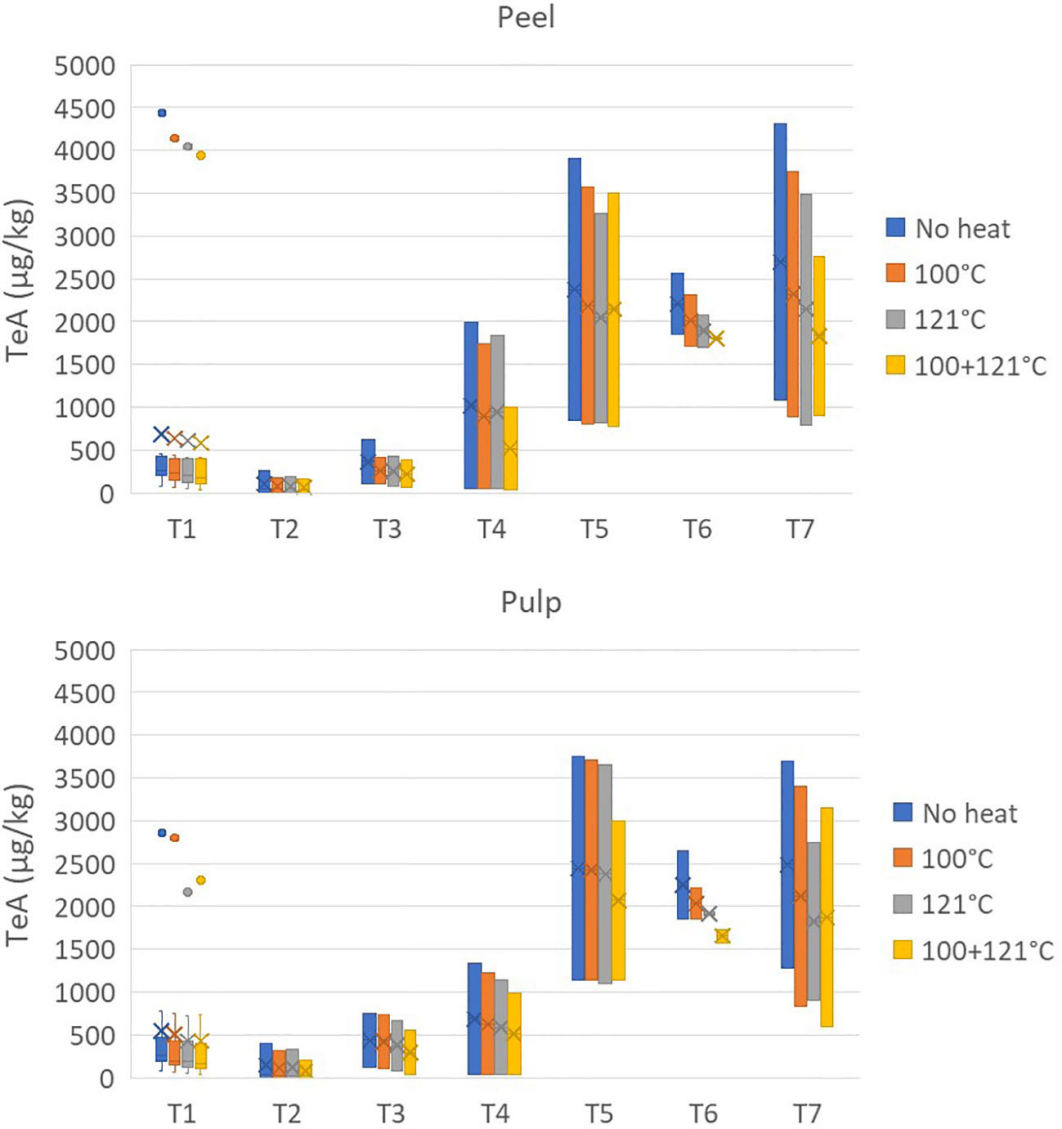
Effect of three different heat treatments on the presence of tenuazonic acid (in micrograms per kilogram) in the peel and pulp of seven different tomato varieties artificially inoculated with *Alternaria* species. Within each box, *horizontal black lines* indicate median values, *crosses* indicate mean values, *boxes* extend from the 25th to the 75th percentile of each group’s distribution of values, the *lower* and *upper lines* denote error lines, and *dots* denote observations outside the 10th and 90th percentiles. The number of samples considered for each tomato variety is reported in **Table 1**.

The effects of heat treatments on TeA are reported in **Figure 4**. TeA showed high stability to heat treatment at 100°C, with average reduction values of 11.23% ± 9.66% and 13.16% ± 10.92% for the peel and pulp, respectively. After treatment at 121°C, the reduction was also not relevant, reaching values of 17.39% ± 10.46% and 20.75% ± 12.83%, while the combined heat treatments (not carried out during tomato processing) decreased the concentration by approximately 30%.

**Figure 5:**
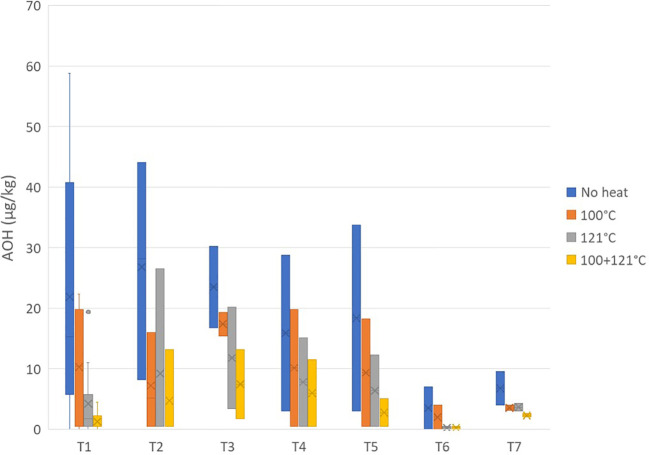
Effect of three different heat treatments on the presence of alternariol (in micrograms per kilogram) in the peel of seven different tomato varieties artificially inoculated with *Alternaria* species. Within each box, *horizontal black lines* indicate median values, *crosses* indicate mean values, *boxes* extend from the 25th to the 75th percentile of each group’s distribution of values, the *lower* and *upper lines* denote error lines, and *dots* denote observations outside the 10th and 90th percentiles. The number of samples considered for each tomato variety is reported in **Table 1**.

The reduction of AOH after heat treatments was considered only for the peel as the presence of this *Alternaria* toxin in the pulp already resulted low prior to the heat treatment (**Figure 5**). Differently from that on TeA, the heat treatment was more effective on AOH, producing a more relevant result: approximately 50% of the toxin was destroyed after treatment at 100°C (average values of 55.34% ± 30.64% for the peel and 42.00% ± 28.01% for the pulp). The reductions increased up to 70% and 80% with treatment at 121°C and with the combined heat treatment, respectively.

**Figure 6:**
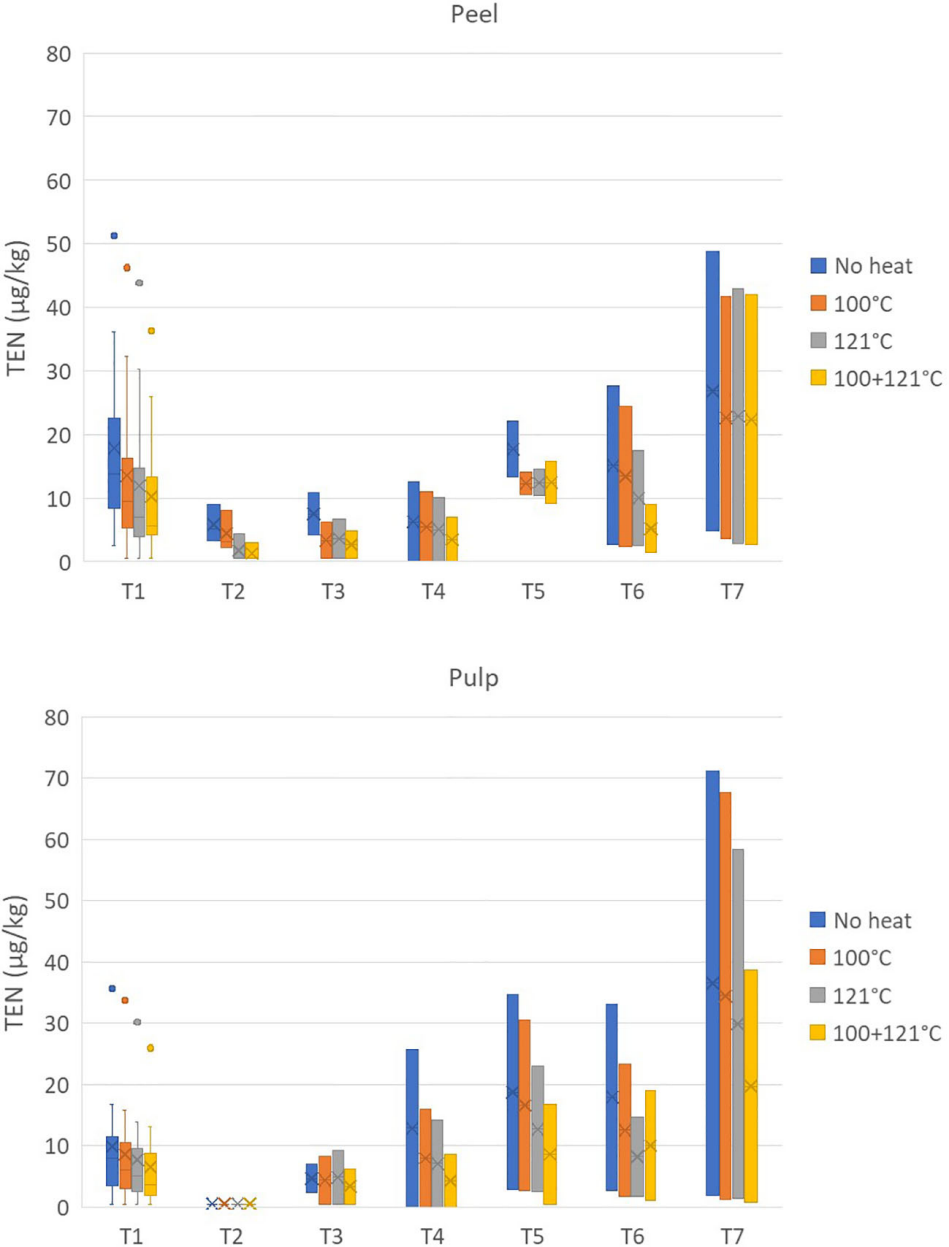
Effect of three different heat treatments on the presence of tentoxin (in micrograms per kilogram) in the peel and pulp of seven different tomato varieties artificially inoculated with *Alternaria* species. Within each box, *horizontal black lines* indicate median values, *crosses* indicate mean values, *boxes* extend from the 25th to the 75th percentile of each group’s distribution of values, the *lower* and *upper lines* denote error lines, and *dots* denote observations outside the 10th and 90th percentiles. The number of samples considered for each tomato variety is reported in **Table 1**.

Finally, the average reductions of TEN after treatment at 100°C were 31.24% ± 21.80% and 22.55% ± 24.75% for the peel and pulp, respectively, and reached values close to 40% and 50% for the 121°C and combined treatments, respectively (**Figure 6**).

The ANOVA results for all of the tomato samples used for the experiment underlined statistically significant differences (*p* ≤ 0.01) due to heat treatments only for AOH (**Table 4**).

**Table 4:** Analysis of variance (ANOVA) of the content of *Alternaria* toxins in the peel and pulp of the 23 tomato samples artificially inoculated with *Alternaria* species after different thermal treatments.

Factor	TeA		AOH				TEN	
	Peel	Pulp	Peel		Pulp		Peel	Pulp
Thermal treatment	n.s.	n.s.	**		**		n.s.	n.s.
Untreated	1,065.4	978.2	18.9	A	2.7	A	14.9	12.2
Treatment at 100°C	995.1	938.3	10.0	AB	0.3	B	12.0	10.9
Treatment at 121°C	949.2	852.6	6.2	B	0.3	B	10.6	9.3
Double treatment (100°C + 121°C)	864.9	785.4	3.1	B	0.3	B	9.0	7.2

Different letters indicate significant differences according to Tukey’s test (*p* < 0.01).

*TeA*, tenuazonic acid; *AOH*, alternariol; *TEN*, tentoxin; *n.s.*, not significant.

**p < 0.01.

## 4 Discussion

Tomato plants can be infected by several pathogens during their cultivation in the field. Among fungi, *Alternaria* spp. have been commonly reported during the tomato growing season and are considered responsible for the visible and severe damages on the plants and fruits, causing important yield losses when environmental conditions are particularly conducive for their development (Nash and Gardner, 1988; Parvin et al., 2021). The infection of tomatoes by *Alternaria* spp., as well as for other fungal infections, is very climate-dependent. To our knowledge, there are no monitoring studies on the presence of *Alternaria* toxins throughout the entire growing season in tomato fields; moreover, the level of mycotoxin contamination in a restricted area can be very different from field to field (Locatelli et al., 2022). For this reason, in order to have different samples with similar infections, an artificial inoculation of tomatoes was preferred and was performed.

The most common *Alternaria* species isolated from Italian tomatoes are *A. solani*, *A. alternata*, and *A. tenuissima* (Garganese et al., 2019; Sanzani et al., 2019). For this reason, it was decided to use isolates belonging to these strains to artificially inoculate samples. Moreover, the strains used have already been tested in previous experiments in order to ensure that they could produce all of the four *Alternaria* toxins reported as important in terms of human exposure and toxicity: AOH, AME, TeA, and TEN (Aichinger et al., 2021; Lin et al., 2023).

Recently, many studies have been conducted in the field and *in vitro* to examine possible methods for containment of *Alternaria* species and *Alternaria* toxins, also considering innovative biological products such as lipopeptides (Zhang et al., 2024), curcumin (Qi et al., 2024), and ginger (Hyder et al., 2024); the use of microorganisms such as bacteria (Bellotti et al., 2023; Bertuzzi et al., 2022; Hyder et al., 2024) or *Trichoderma* species (Bassant et al., 2024); and the use of new technologies such as the nanosystem approach (Khatoon et al., 2024; Xu et al., 2024), demonstrating the emerging interest linked to this fungal pathology able to produce mycotoxins in tomato products worldwide (Bertuzzi et al., 2021; Xu et al., 2024) and with possible health risks to consumers. The results obtained from this study underlined a different distribution of *Alternaria* toxins in tomato peel and pulp.

In particular, the findings of this work can indicate that the widespread occurrence of TeA in tomato-based products is certainly due to the high contamination in raw moldy tomatoes; however, it can also be favored by migration from the peel, where the fungal contamination starts in the field and is normally considered as a by-product, to the pulp. The different distribution of the *Alternaria* toxins in the peel and pulp of the tomato samples could be explained by their different chemical properties. Unlike AOH and TEN, TeA is a monobasic acid with p*K*_a_ = 3.5 (EFSA, 2011), which can then easily move in the acidic water-rich pulp fraction of tomatoes. On the other hand, the limited occurrence of AOH in tomato products can also be due to its limited migration in the pulp, remaining in the skin waste.

This study has some limitations due to not being a complete replication of field conditions. In particular, as we wanted to achieve a very high contamination in tomatoes, we used a high concentration of *Alternaria* spores for the preparation of the artificial inoculum, a concentration normally never present in the field. However, as we wanted to have a high amount of mycotoxins, a high level of inoculum was necessary. Tomatoes were also damaged in order to favor the fungal infection and make it more speedy; this parameter is also not always present in the field, where fruits can also be contaminated without any damage. Moreover, detached tomatoes were used without consideration of the possible role of the plant in disease containment. However, the results obtained in this study can be considered preliminary, but also very important in order to determine the distribution of the different *Alternaria* toxins in fruits to better define possible measures for their containment during tomato transformation.

Among the *Alternaria* toxins present in the water after the incubation of tomatoes, only AOH was never detected. TeA and TEN have been reported to be easily removed from the tomato surface through washing (Estiarte et al., 2018). However, the water taken into consideration was not derived from the tomato washing, but is distilled and sterilized water placed inside the glass boxes for maintenance of high humidity. Furthermore, the water was not in contact with the tomatoes. However, during the incubation time (3 weeks), the tomatoes decayed, releasing little quantity of liquid that contaminated the water. It is interesting that the TeA levels in the water were very high, indicating that its hydrophilic properties can favor migration in water solutions. This trend could indicate that the water used for tomato washing and/or for fruit movement during the industrial processing can become contaminated if not frequently replaced or when not using large volumes of water. The high mobility of TeA in the pulp and in aqueous solutions may facilitate cross-contamination from a limited number of highly contaminated tomatoes to whole derived products, obtained from different lots of tomatoes processed simultaneously.

Similarly to other mycotoxins, *Alternaria* toxins are not completely destroyed during heat processing. Siegel et al. (2010) reported a limited degradation of AOH, AME, and altenuene during wet bread baking, but a significant degradation upon dry baking. Estiarte et al. (2018) reported that heating of tomato samples at 100°C and 110°C significantly affected the stability of AOH. Our results confirmed this last study. Approximately 50% of AOH was destroyed after heat treatments at conditions close to those used in industrial tomato processing. AOH belongs to the family of isocoumarins and derivatives. To our knowledge, there is no information available on the thermal stability of AOH in different matrices. It is probable that both the acidic conditions of the matrix (the pH of tomato is approximately 3.5) and the high temperature, as well as the limited migration into the pulp (in our experiments, the inner temperature of tomatoes was lower than that in the peel; data not shown), contribute to the reduction in AOH. It will be important to recognize its metabolites and their toxicity. The reduction of TeA and TEN contamination was extremely limited, showing high stability to heat treatments. The best results were obtained with the double heat treatment (100°C + 121°C). However, this type of treatment is not used in industrial tomato processing as it greatly worsens the quality of the final products, modifying the color and decreasing the nutrient content.

The findings of this work improve our understanding of the behavior of Alternaria species during the infection phase and in the definition of protocols useful for reducing the presence of *Alternaria* toxins in tomato-derived products. In particular, the different distribution of toxins in the pulp and the skin during the infection cycle of the fungus needs to be investigated further, while the washing and the sorting steps appear to be two of the most important points during tomato processing.

The use of clean and abundant water can help in reducing the level of certain mycotoxins such as TeA and TEN in the final product, while the removal of visibly defective fruits could be essential in reducing the initial contamination levels of tomatoes as heat treatments are not able to greatly reduce the presence of *Alternaria* toxins in the final derived products. Indeed, the EU Recommendation 2022/553 reported a single indicative limit for all tomato-derived products, independently of the concentration factor occurring during the processing with respect to raw tomatoes. It will be important to evaluate both the concentration factor of the different final products (i.e., sauce, pulp, and paste) and the different values of migration in the pulp and heat stability for each *Alternaria* toxin, as reported in this study.

Further studies are needed to understand the critical control points of tomato processing and possible corrective measures to reducing the risk of contamination of the final products and protecting consumers’ health.

## Data Availability

The original contributions presented in the study are included in the article/**Supplementary Material**. Further inquiries can be directed to the corresponding author.
