# Supplementary material for: Distribution of Alternaria toxins in tomato pulp and peel and their stability to heat treatments
Source: Front Fungal Biol. 2025 Jun 6;6:1516557. doi: 10.3389/ffunb.2025.1516557 (PMC12179057; doi:10.3389/ffunb.2025.1516557)

Supplementary Material

Supplementary Table 1. Calibration curve of tenuazonic acid (TeA).

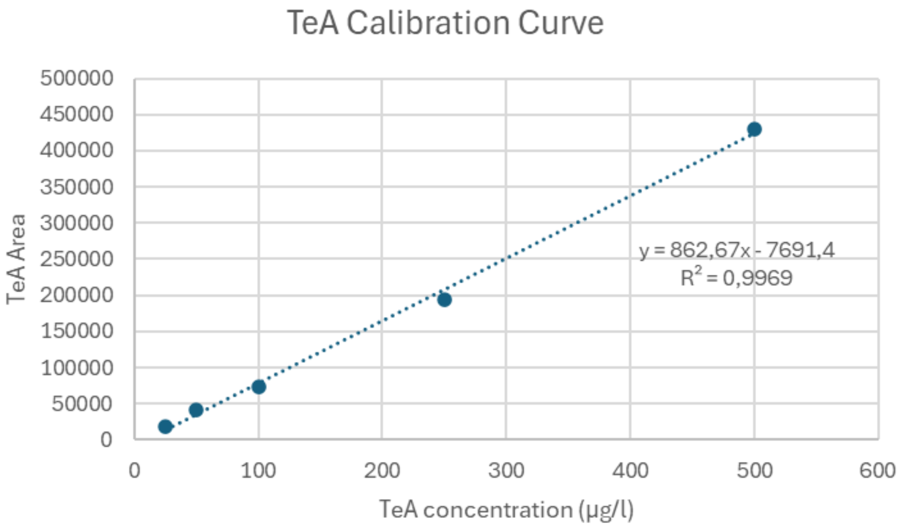

Supplementary Table 2. Tenuazonic acid concentration (μg/kg) in pulp and peel of the 23 tomato samples artificially inoculated with *Alternaria* strains.

**Sample code**	**TeA in pulp (μg/kg)**	**TeA in peel (μg/kg)**
S1	131	223
S2	30	55
S3	< LOD	< LOD
S4	749	626
S5	226	126
S6	122	107
S7	405	254
S8	206	229
S9	71	71
S10	324	278
S11	780	422
S12	36	52
S13	1132	846
S14	2641	2563
S15	238	223
S16	3691	4309
S17	273	410
S18	1853	1849
S19	2858	4438
S20	1284	1085
S21	3753	3899
S22	362	455
S23	1334	1984

Supplementary Table 3. Alternariol concentration (µg/kg) in pulp and peel of the 23 tomato samples artificially inoculated with *Alternaria* strains.

**Sample code**	**AOH in pulp (µg/kg)**	**AOH in peel (µg/kg)**
S1	4.4	58.8
S2	0.5	28.0
S3	3.0	44.0
S4	2.7	17.0
S5	5.0	36.0
S6	4.0	30.0
S7	0.5	8.0
S8	8.9	22.0
S9	8.0	54.9
S10	< LOD	< LOD
S11	0.5	5.0
S12	0.5	3.0
S13	0.5	3.0
S14	< LOD	< LOD
S15	0.5	9.0
S16	0.5	4.0
S17	3.0	6.0
S18	0.5	7.0
S19	0.5	6.0
S20	4.9	9.6
S21	6.0	33.8
S22	0.5	21.7
S23	6.3	28.7

Supplementary Table 4. Tentoxin concentration (µg/kg) in pulp and peel of the 23 tomato samples artificially inoculated with *Alternaria* strains.

Sample code	TEN in pulp (µg/kg)	TEN in peel (µg/kg)
S1	0.5	9.2
S2	0.5	5.2
S3	0.5	9.0
S4	6.9	10.9
S5	4.0	7.9
S6	2.4	4.2
S7	0.5	3.3
S8	4.8	17.6
S9	9.7	11.3
S10	7.0	8.6
S11	1.7	2.5
S12	25.7	12.6
S13	34.7	22.2
S14	2.7	2.6
S15	9.3	18.0
S16	1.9	4.9
S17	16.8	36.1
S18	33.1	27.6
S19	35.7	51.2
S20	71.1	48.8
S21	2.8	13.3
S22	8.9	16.3
S23	< LOD	< LOD

Supplementary Figure 1. Tenuazoic acid (TeA) concentration (µg/kg) in peel (Graph 1.A) and pulp (Graph 1.B) of the 23 tomato samples artificially inoculated with *Alternaria* strains before and after heat treatments.

1.A)

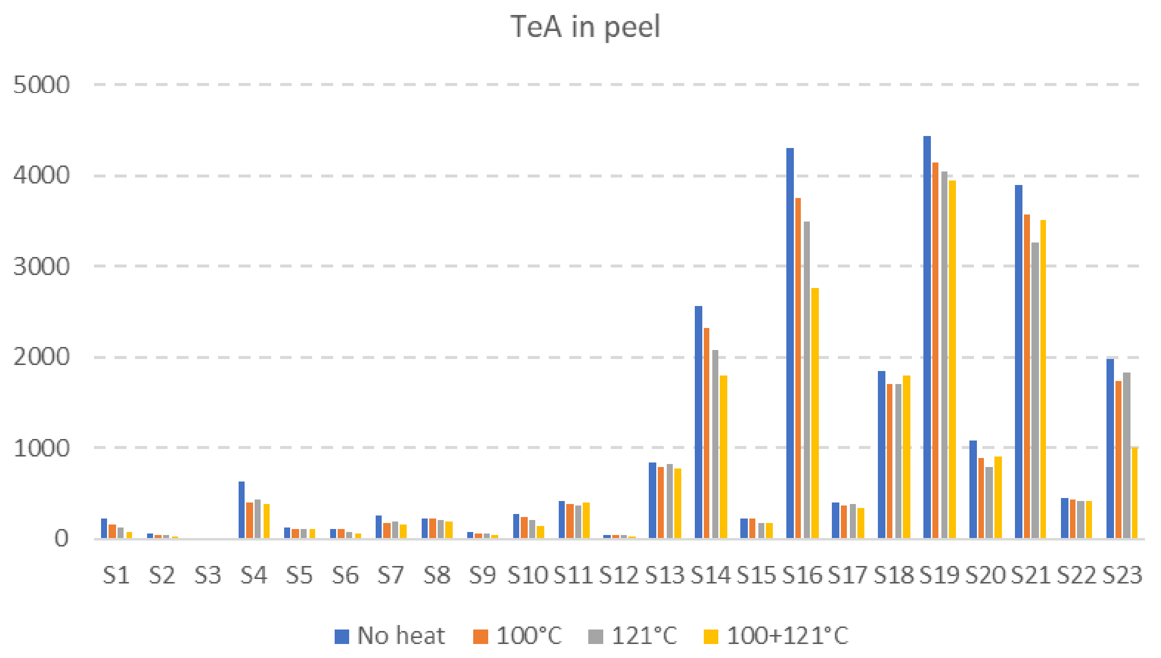

1.B)

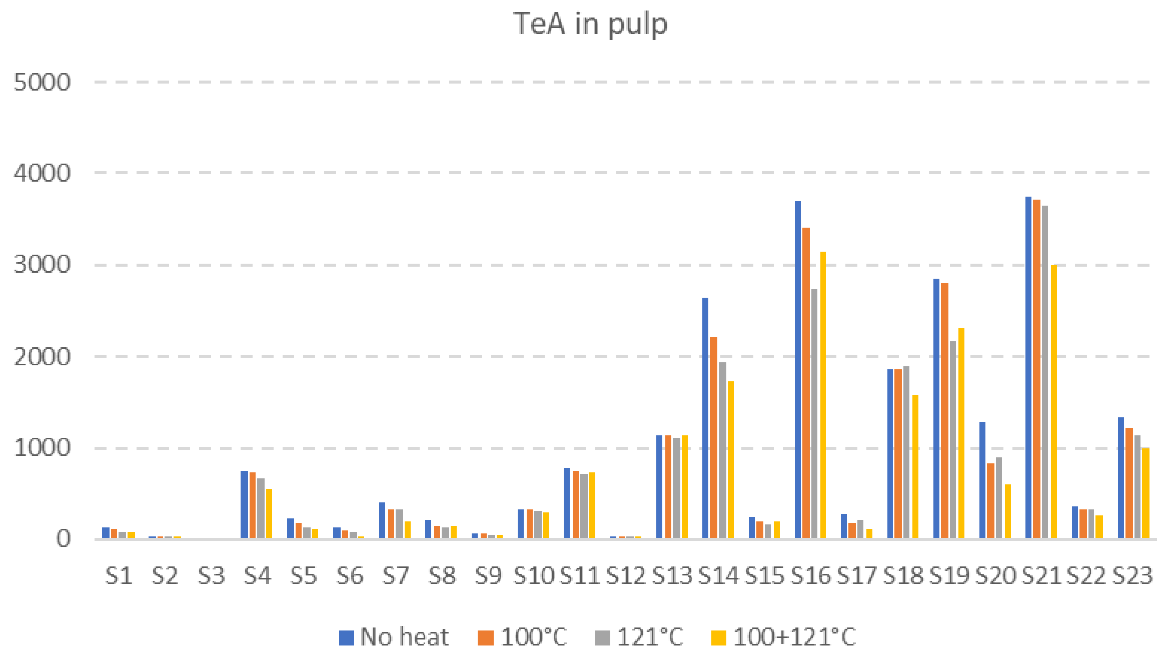

Supplementary Figure 2. Alternariol (AOH) concentration (µg/kg) in peel of the 23 tomato samples artificially inoculated with *Alternaria* strains before and after heat treatments.

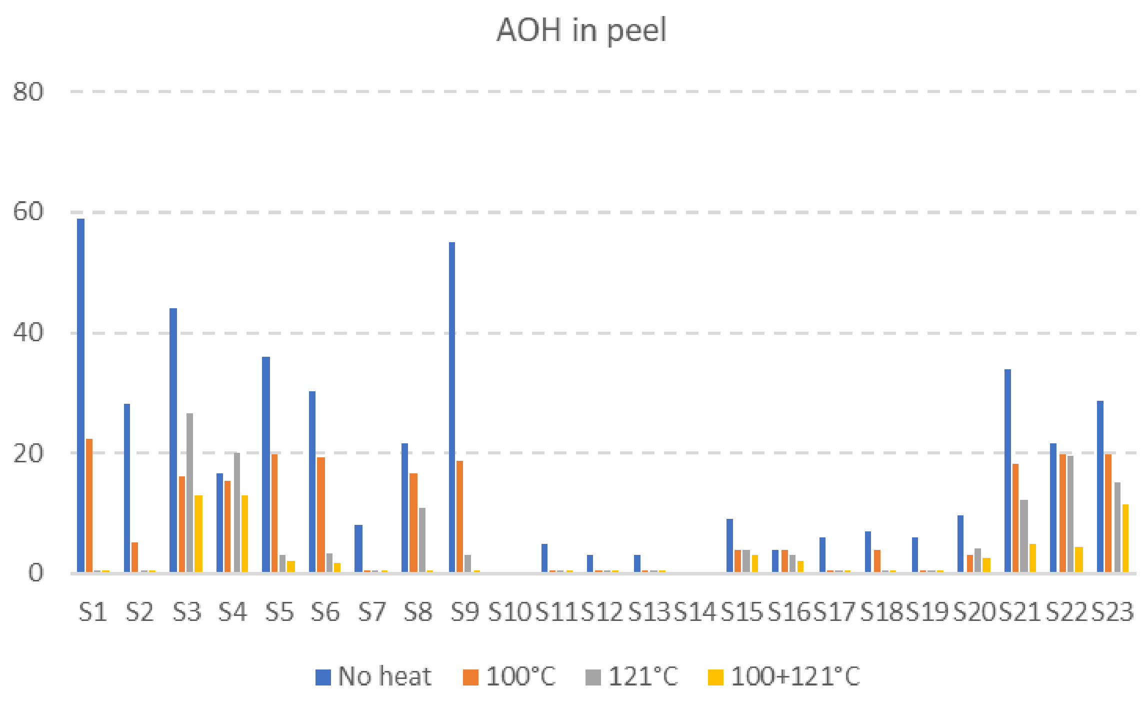

Supplementary Figure 3. Tentoxin (TEN) concentration (µg/kg) in peel (Graph 3.A) and pulp (Graph 3.B) of the 23 tomato samples artificially inoculated with *Alternaria* strains before and after heat treatments.

3.A)

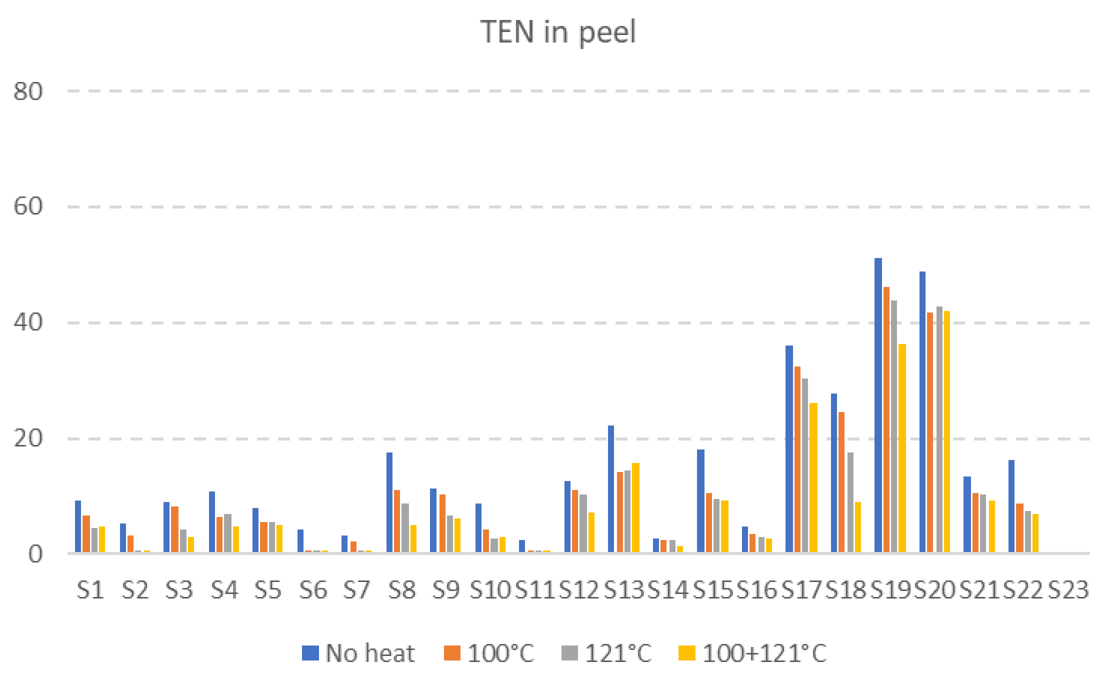

3.B)

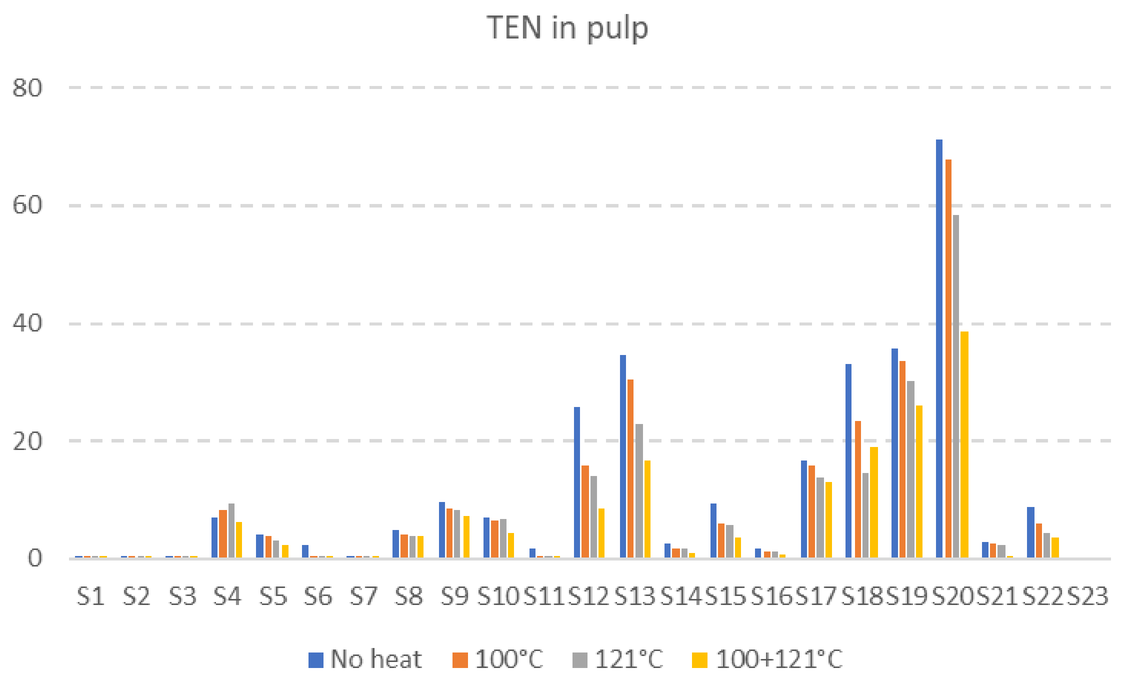

Supplement: Supplementary file 1 [file DataSheet1.pdf]
